# Patient- and Surgery-Related Factors that Affect Patient-Reported Outcomes after Total Hip Arthroplasty

**DOI:** 10.3390/jcm7100358

**Published:** 2018-10-15

**Authors:** Toshiyuki Kawai, Masanao Kataoka, Koji Goto, Yutaka Kuroda, Kazutaka So, Shuichi Matsuda

**Affiliations:** Department of Orthopedic Surgery, Kyoto University Graduate School of Medicine, 54 Shogoin-Kawaharacho, Sakyo-ku, Kyoto 606-8507, Japan; masarun_run_42195@yahoo.co.jp (M.K.); k.g.bau@kuhp.kyoto-u.ac.jp (K.G.); ykuromd@gmail.com (Y.K.); sokazu71@gmail.com (K.S.); smat522@kuhp.kyoto-u.ac.jp (S.M.)

**Keywords:** total hip arthroplasty, Oxford Hip Score, Harris Hip Score, patient reported outcome measure, UCLA activity score

## Abstract

Patient-reported outcome measures (PROMs) are used to assess satisfaction after total hip arthroplasty (THA); however, the factors that determine these PROMs remain unclear. This study aimed to identify the patient- and surgery-related factors that affect patient satisfaction after THA as indicated by the Oxford Hip Score (OHS). One-hundred-and-twenty patients who underwent primary THA were included. Various patient-related factors, including clinical scores, and surgery-related factors were examined for potential correlations with the OHS at 3, 6, and 12 months post-THA. Univariate regression analysis showed that higher preoperative University of California Los Angeles (UCLA) activity score (*p* = 0.027) and better preoperative OHS (*p* = 0.0037) were correlated with better OHS at 3 months post-THA. At 6 months post-THA, the factors associated with better OHS were higher preoperative UCLA activity score (*p* = 0.039), better preoperative OHS (*p* = 0.0006), and use of a cemented stem (*p* = 0.0071). At 12 months post-THA, the factors associated with better OHS were higher preoperative UCLA activity score (*p* = 0.0075) and better preoperative OHS (*p* < 0.0001). Multivariate regression analysis showed that the factors significantly correlated with better OHS were female sex (*p* = 0.011 at 3 months post-THA), osteoarthritis (*p* = 0.043 at 6 months), higher preoperative OHS (*p* < 0.001 at 3 and 12 months, *p* = 0.018 at 6 months), higher preoperative Harris Hip Score (*p* = 0.001 at 3 months), higher preoperative UCLA activity score (*p* = 0.0075 at 3 months), and the use of a cemented femoral component (*p* = 0.012 at 6 months). Patient- and surgery-related factors affecting post-THA PROMs were identified, although the effect of these factors decreased over time.

## 1. Introduction

Total hip arthroplasty (THA) is effective in relieving pain and improving function in osteoarthritic hips [1]. Hence, more than 200,000 THAs are performed in the USA each year [2]. The success of a THA has been assessed via several methods. Most studies have determined the success of THA via implant survival rates and implant-related complications; however, there is an increasing interest in patient-reported outcome measures (PROMs) [3]. Patient-reported outcome scores are the most common method used to quantify the results of treatments that aim to improve pain, stiffness, function, and quality of life. PROMs after THA are reportedly affected by sex, age, diagnosis, anxiety/depression, pain, and function [4,5,6,7,8,9,10].

One of the most common PROMs used to assess the outcome of THA is the Oxford Hip Score (OHS). The OHS has been used in many clinical trials since its introduction in 1996, and has proven reliability and validity [11,12]. Furthermore, the OHS is strongly correlated with the Harris Hip Score (HHS) before and after hip surgery [13,14]. One of the most important outcomes in THA is satisfaction with the surgery, and the percentage of patients dissatisfied with the results of THA is reportedly small [15,16]. While it has been reported that PROMs may be correlated with some patient-related factors such as sex, age, and diagnosis, data regarding the relationship between PROMs and surgery-related factors are limited.

The aim of the present study was to determine which patient- and surgery-related factors affect the OHS after THA.

## 2. Materials and Methods

This was a prospective cohort study. All patients provided informed consent, and the study protocol was approved by the Institutional Review Board of our hospital. Between July 2014 and July 2016, we performed 200 primary THAs in 183 patients. Among them, 78 were excluded from the present study because the condition of the contralateral hip may have affected the clinical and satisfaction scores. Specifically, the contralateral hips of the excluded patients had untreated osteonecrosis of the femoral head (ONFH) (*n* = 15), severe osteoarthritis (OA) (*n* = 39), and a total hip joint implanted within 12 months before the index surgery (*n* = 24). Another two hips were excluded because the procedure involved subtrochanteric shortening osteotomy, which could be associated with longer rehabilitation and additional risk of nonunion. This left 120 procedures in 120 patients whose contralateral hip was either normal or had undergone a THA more than 1 year ago.

All 120 procedures were carried out by a senior hip surgeon (K.G., K.S., or Y.K.). All surgeries were performed via the conventional anterolateral approach or the minimally invasive anterolateral approach [17]. On the femoral side, a cemented stem was used in 89 hips, while an uncemented stem was used in 31 hips. On the acetabular side, a cemented cup was used in five hips, while an uncemented cup was used in 115 hips. The implant choice was made depending on the surgeon’s preference. The cemented stem was the H3 taper (Kyocera Medical, Kyoto, Japan) in 87 hips, and the Kyocera Type 6 (Kyocera Medical) in two hips. The uncemented stem was the Anthology (Smith & Nephew, Boston, MA, USA) in two hips, the J-taper (Kyocera Medical) in five hips, the SL-PLUS (Smith & Nephew) in 22 hips, and the Taperloc (Zimmer Biomet, Warsaw, IN, USA) in two hips. The cemented cup was the GP-FL (Kyocera Medical) in four hips, and the CLHO (Kyocera Medical) in one hip. The uncemented cup was the AHFIX (Kyocera Medical) in 91 hips, the R3 (Smith & Nephew) in five hips, and the REFLECTION (Smith & Nephew) in 19 hips.

Demographic data of the included patients are shown in Table 1, including age, gender, body mass index (BMI), diagnosis, Crowe classification [18], duration of surgery, pre- and post-operative leg length discrepancy (LLD), and component type (cemented or uncemented) used for the index THA.

The leg length discrepancy (LLD) was measured by comparing the distance between the most prominent point of the lesser trochanter and the inter-teardrop line on anterior-posterior radiographs of both hips before versus immediately after THA [19].

Clinical assessments were performed using the HHS, the UCLA activity score, and the OHS preoperatively and at 3, 6, and 12 months postoperatively. The calculation of the OHS was performed according to the original scoring system, where each question was scored from 1 to 5, with 1 representing best outcome or least symptoms. Overall score was from 12 to 60 with 12 being the best outcome. The clinical examination and data collection for the HHS and the UCLA activity score were done by one of the surgeons, but the OHS questionnaire was handed out and collected by hospital staff rather than the surgeon, so that patients could give their honest opinions even if they were not satisfied with the surgery.

We considered the following patient- and surgery-related covariates: Age, sex, body mass index (BMI), preoperative diagnosis, Crowe group, preoperative LLD, postoperative LLD, preoperative UCLA activity score, preoperative OHS, preoperative HHS, range of motion (ROM) in flexion, duration of surgery, and the use of a cemented or uncemented component on the acetabular and femoral sides.

### Statistical Analysis

Differences in proportions were calculated by the chi-squared test. Differences in means were calculated by the Wilcoxon test for the comparison of two groups, or by the Kruskal-Wallis test followed by the post hoc Steel-Dwass test for the comparison of more than two groups. Probability values < 0.05 were considered significant. Univariate regression using the OHS at each timepoint as a dependent variable was performed when the independent variable was a continuous variable. Multivariate regression analysis was performed using the stepwise regression model with the OHS at each timepoint as a dependent variable, and age, sex, BMI, preoperative diagnosis, Crowe group, preoperative LLD, postoperative LLD, preoperative UCLA activity score, preoperative OHS, preoperative HHS, ROM in flexion, surgery time, and the use of a cemented or uncemented component on the acetabular and femoral sides as the independent variables. All statistical analyses were carried out using JMP Pro 14 software (SAS Institute, Cary, NC, USA).

## 3. Results

The OHS, HHS and UCLA activity score at each time point were shown in Table 2. The average LLD was 11.6 (0–53.3) mm preoperatively, and 4.0 (0–29.1) mm after THA as shown in Table 1. A significantly greater proportion of patients had an LLD of less than 10 mm after THA (113 (94.2%)) compared with preoperatively (67 (55.8%); *p* = 0.0026). The ROM in flexion was 81 ± 21.1° preoperatively, 91.9 ± 13.3° at 3 months post-THA, 92.6 ± 13.7° at 6 months post-THA, and 94.9 ± 13.7° at 12 months post-THA.

Table 3, Table 4, Table 5 and Table 6 show the OHS, HHS, and UCLA activity score at each timepoint for each sex, diagnosis group, Crowe group, and type of acetabular and femoral component. Male patients had a slightly better mean preoperative OHS, preoperative HHS, and preoperative UCLA activity score than female patients; however, those differences were not significant (*p* = 0.16, *p* = 0.22, and *p* = 0.069, respectively). Patients diagnosed with OA had a significantly better preoperative OHS than patients with ONFH (*p* = 0.010), but there was no significant difference in preoperative OHS between patients with OA versus those with RDC (*p* = 0.23). There was no significant difference in preoperative HHS and preoperative UCLA activity score between patients with ONFH versus those with RDC. Comparisons between Crowe group I versus groups II to IV did not show any significant differences in preoperative OHS, preoperative HHS, and preoperative UCLA activity score.

**Figure 1:**
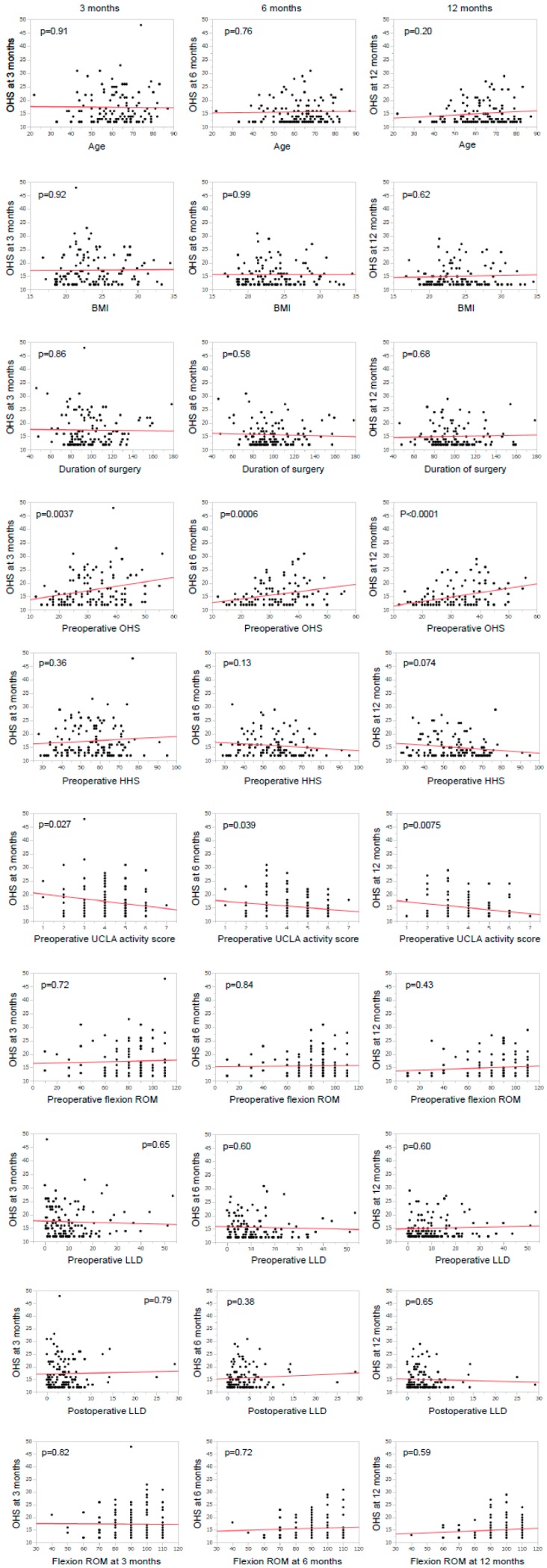
Univariate regression analysis of the relationships between each variable and the Oxford Hip Score (OHS) at 3, 6, 12 months after total hip arthroplasty. The solid lines indicate the regression lines. BMI: Body mass index; HHS: Harris Hip Score; ROM: Range of motion; LLD: Leg length discrepancy; UCLA: University of California Los Angeles.

The relationships between postoperative OHS and age, BMI, duration of surgery, preoperative OHS, preoperative HHS, preoperative UCLA activity score, flexion ROM, preoperative LLD, and postoperative LLD examined by univariate regression analysis are illustrated in Figure 1. Univariate regression analysis demonstrated that a lower OHS at 3 months post-THA was significantly associated with higher preoperative UCLA activity score (*p* = 0.027) and lower preoperative OHS (*p* = 0.0037); the use of a cemented cup and the use of an uncemented stem tended to result in a higher OHS, but these correlations were not significant (*p* = 0.091 and *p* = 0.075, respectively). A lower OHS at 6 months post-THA was significantly correlated with higher preoperative UCLA activity score (*p* = 0.039), lower preoperative OHS (*p* < 0.001), and the use of a cemented femoral component (*p* < 0.01). A lower OHS at 12 months post-THA was significantly correlated with higher preoperative UCLA activity score (*p* < 0.01) and lower preoperative OHS (*p* < 0.001).

In a multivariate model, as shown in Table 7, a lower OHS at 3 months post-THA was significantly correlated with the female sex (|*r*| = 0.19, *p* < 0.01), higher HHS (|*r*| = 0.51, *p* < 0.01), higher preoperative UCLA activity score (|*r*| = 0.48, *p* < 0.01), and lower preoperative OHS (|*r*| = 0.45, *p* < 0.001). A lower OHS at 6 months post-THA was significantly correlated with age (|*r*| = 0.45, *p* < 0.05), lower preoperative OHS (|*r*| = 0.31, *p* < 0.01), and the use of a cemented femoral component. A lower OHS at 12 months post-THA was only significantly associated with lower preoperative OHS (|*r*| = 0.19, *p* < 0.001).

## 4. Discussion

Patients generally show high levels of satisfaction with THA outcomes. However, PROMs after THA are reportedly affected by sex, age, diagnosis, anxiety/depression, pain, and function [5,6,7,8,9,10]. In the present study, although females had a significantly better OHS at 3 months post-THA than males, the correlation between female sex and better OHS was weak and became non-significant at 6 and 12 months post-THA.

Patients treated for OA generally had better postoperative OHS than patients with ONFH or RDC, and multivariate regression analysis showed that this difference was significant at 6 months post-THA. In contrast, there was little difference between diagnosis groups in the HHS at the same timepoint.

Older age is reportedly a predictor of a poor PROM score [8]. Our results also demonstrated that older patients tended to have a poorer OHS than younger patients at 3 and 6 months post-THA, but these differences were not significant.

Although higher BMI is reportedly associated with a low function score [20] and poor PROM score [21], we did not find any correlation between BMI and postoperative OHS. Similarly, Liu et al. [20] reported that although patients with obesity had a significantly lower HHS than non-obese patients, the OHS did not significantly differ between those with versus without obesity. However, the present study only included eight patients (6.7%) with a BMI of more than 30 kg/m^2^, and no patient had a BMI of more than 35 kg/m^2^. Hence, the present results cannot be applied for THA outcome prediction in patients with a BMI of more than 35 kg/m^2^.

Some authors report that LLD is not correlated with the clinical score [22,23]; however, others report that patients with an LLD of more than 1 cm are often aware of the inequality and are disturbed by it [24,25]. Our results did not show any significant correlation between patient satisfaction and LLD before or after THA. However, the LLD was less than 10 mm in 113 patients, and was 10 mm or more in only seven; this may be too few cases to enable us to draw a conclusion on the effect of LLD on postoperative OHS. In general, we try to minimize the LLD as much as the muscle condition allows. It is also important to be aware that LLD is the second-most common reason for litigation after THA [26].

The duration of surgery could represent the complexity of the procedure. Therefore, there was a concern that longer surgery may result in a lower satisfaction score. However, the present study found no correlation between duration of surgery and satisfaction after THA. The present cohort did not experience any major intraoperative complications such as fracture, which may explain the lack of correlation between longer surgical duration and lower satisfaction.

In the present study, preoperative OHS was a predictor of good OHS at 3, 6, and 12 months post-THA. It is logical that patients who already have a good OHS before THA are likely to have a good OHS after surgery, as the same measurement system is used. Although better preoperative OHS was associated with better postoperative OHS in this study, this does not mean that patients with better hip function are the better candidates for THA. Since THA is effective in relieving pain and improving function, the indication of THA should be the hip pain and poor hip function.

Higher preoperative HHS and better preoperative UCLA activity score were also predictors of better OHS at 3 months post-THA. However, these scores were no longer correlated with OHS at 6 and 12 months post-THA. Patients with lower function or lower activity before THA tended to show low levels of satisfaction in the early postoperative phase, but this tendency disappeared over time. In the present study, patients who underwent THA generally had a good OHS at 12 months postoperatively, regardless of preoperative function or preoperative activity, although preoperative OHS was still a predictor of OHS at 12 months post-THA.

We also examined the effect of preoperative flexion ROM and postoperative flexion ROM on OHS after THA, and found that neither the pre- nor postoperative ROM affected the postoperative satisfaction score.

The present study evaluated the effect of patient- and surgery-related factors on postoperative patient-reported outcome. Female sex, diagnosis of OA, use of a cemented stem, better preoperative OHS, better preoperative HHS, and better preoperative UCLA activity score were associated with better postoperative OHS during the early follow-up period, but the effects of these variables tended to disappear by 1 year postoperatively, except for preoperative OHS. Most of the surgery-related factors (such as postoperative LLD, duration of surgery, and flexion ROM at follow-up) did not show any correlation with postoperative OHS. Prospective and randomized studies are needed to provide more information on the relationship between those surgery-related factors and PROMs.

## Figures and Tables

**Table 1:** Patient demographic characteristics.

Variable	Average (Range) or Percentage
Age	62.4 (22–87)
Gender	
Male	30 (25%)
Female	90 (75%)
BMI	23.7 (16.8–34.5)
Diagnosis	
OA	94 (78%)
ONFH	22 (18%)
RDC	4 (4%)
Crowe classification	
Group I	106 (88%)
Group II	10 (8%)
Group III	1 (1%)
Group IV	3 (3%)
Duration of surgery (min)	99.5 (46–178)
Pre-op leg length discrepancy (absolute value) (mm)	11.6 (0–53.3)
Post-op leg length discrepancy (absolute value) (mm)	4.0 (0–29.1)
Acetabular component	
Cemented	5 (4.2%)
Uncemented	115 (95.8%)
Femoral component	
Cemented	31 (25.8%)
Uncemented	89 (74.2%)

Data are shown as the number (percentage) or average (minimum–maximum). BMI: Body mass index; OA: Osteoarthritis; ONFH: Osteonecrosis of femoral head; RDC: Rapidly destructive coxarthrosis.

**Table 2:** Clinical scores preoperatively, and at 3, 6, and 12 months postoperatively.

Time Point	OHS	HHS	UCLA Activity Score
Pre-op	31.3 ± 9.4 (12–56)	55.6 ± 13.5 (28–95)	4.1 ± 1.3 (1–7)
3 months	17.4 ± 5.9 (12–48)	89.1 ± 9.8 (50–100)	5.1 ± 1.0 (2–8)
6 months	15.7 ± 4.2 (12–31)	92.5 ± 7.5 (72–100)	5.5 ± 1.1 (2–8)
12 months	15.0 ± 4.0 (12–29)	94.0 ± 7.0 (73–100)	5.4 ± 1.1 (2–9)

Data are shown as the average ± standard deviation (minimum–maximum). OHS: Oxford Hip Score; HHS: Harris Hip Score; UCLA: University of California Los Angeles.

**Table 3:** Preoperative clinical scores.

Variable		OHS Score	*p* Value	HHS Score	*p* Value	UCLA Activity Score	*p* Value
Sex	Male	29.2 ± 7.8	0.16	58.0 ± 13.1	0.22	4.4 ± 1.3	0.069
	Female	32.0 ± 9.8		54.8 ± 13.6		4.0 ± 1.2	
Diagnosis	OA	29.8 ± 9.0	0.0054 *	56.3 ± 13.4	0.66	4.1 ± 1.3	0.19
	ONFH	36.2 ± 8.5		53.2 ± 13.1		4.0 ± 1.3	
	RDC	39.0 ± 11.6		53.8 ± 18.9		2.8 ± 1.5	
Crowe classification	Group I	31.4 ± 9.5	0.72	56.2 ± 13.0	0.28	4.1 ± 1.3	0.52
	Group II, III, IV	30.4 ± 9.0		52.1 ± 15.8		3.9 ± 1.2	
Acetabular component	Cemented	27.8 ± 10.0	0.46	69.0 ± 17.4	0.15	4.3 ± 1.7	0.81
	Uncemented	31.4 ± 9.4		55.3 ± 13.1		4.1 ± 1.3	
Femoral component	Cemented	30.6 ± 9.3	0.17	54.7 ± 14.0	0.17	4.0 ± 1.3	0.10
	Uncemented	33.3 ± 9.5		58.5 ± 11.0		4.4 ± 1.2	

Scores are shown as the average ± standard deviation. The *p* value for the comparison of the diagnosis groups was obtained by the Kruskal-Wallis test. The *p* values for all other comparisons were obtained by the Wilcoxon test. * There was a significant difference between OA and ONFH (*p* = 0.013, Steel-Dwass test). OHS: Oxford Hip Score; HHS: Harris Hip Score; OA: Osteoarthritis; ONFH: Osteonecrosis of the femoral head; RDC: Rapidly destructive coxarthrosis. UCLA: University of California Los Angeles.

**Table 4:** Clinical scores 3 months after total hip arthroplasty.

Variable		OHS Score	*p* Value	HHS Score	*p* Value	UCLA Activity Score	*p* Value
Sex	Male	18.9 ± 7.6	0.11	87.3 ± 12.2	0.20	5.2. ± 1.0	0.75
	Female	16.9 ± 5.2		89.6 ± 8.9		5.1 ± 1.0	
Diagnosis	OA	16.7 ± 5.5	0.05 ^#^	89.5 ± 9.2	0.57	5.2 ± 1.0	0.66
	ONFH	19.7 ± 7.0		87.7 ± 12.1		5.2. ± 0.9	
	RDC	20.3 ± 4.6		85.3 ± 10.1		4.8 ± 1.0	
Crowe classification	Group I	17.3 ± 6.1	0.69	89.9 ± 9.1	0.01	5.2 ± 1.0	0.02
	Group II, III, IV	18.0 ± 4.3		83.1 ± 13.0		4.5 ± 1.1	
Acetabular component	Cemented	18.5 ± 2.6	0.28	81.0 ± 21.3	0.032	4.5 ± 2.1	0.087
	Uncemented	17.3 ± 5.9		89.5 ± 9.1		5.2 ± 1.0	
Femoral component	Cemented	16.8 ± 5.7	0.064	89.8 ± 9.2	0.18	5.1 ± 1.1	0.78
	Uncemented	19.1 ± 6.3		87.1 ± 11.0		5.2 ± 0.7	

Scores are shown as the average ± standard deviation. The *p* value for the comparison of the diagnosis groups was obtained by the Kruskal-Wallis test. The *p* values for all other comparisons were obtained by the Wilcoxon test. ^#^ The post hoc Steel-Dwass test showed that there was no significant difference between any two groups. OHS: Oxford Hip Score; HHS: Harris Hip Score; OA: Osteoarthritis; ONFH: Osteonecrosis of the femoral head; RDC: Rapidly destructive coxarthrosis; UCLA: University of California Los Angeles.

**Table 5:** Clinical scores 6 months after total hip arthroplasty.

Variable		OHS Score	*p* Value	HHS Score	*p* Value	UCLA Activity Score	*p* Value
Sex	Male	15.9 ± 4.1	0.74	92.1 ± 7.3	0.87	5.6 ± 1.1	0.48
	Female	15.6 ± 4.2		92.6 ± 7.6		5.5 ± 1.1	
Diagnosis	OA	15.1 ± 3.4	0.13	92.5 ± 7.5	0.82	5.6 ± 1.2	0.23
	ONFH	18.0 ± 6.1		93.0 ± 7.7		5.5 ± 0.8	
	RDC	16.8 ± 3.0		90.5 ± 7.5		4.5 ± 1.3	
Crowe classification	Group I	15.6 ± 4.0	0.55	93.4 ± 7.1	<0.001	5.6 ± 1.9	0.037
	Group II, III, IV	16.3 ± 5.4		85.3 ± 6.0		4.9 ± 1.1	
Acetabular component	Cemented	16.5 ± 4.8	0.34	89.3 ± 10.3	0.16	4.8 ± 2.2	0.061
	Uncemented	15.6 ± 4.1		92.6 ± 7.3		5.5 ± 1.1	
Femoral component	Cemented	15.1 ± 3.9	0.012	92.5 ± 7.5	0.80	5.5 ± 1.2	0.82
	Uncemented	17.3 ± 4.4		92.1 ± 7.5		5.5 ± 0.9	

Scores are shown as the average ± standard deviation. The *p* value for the comparison of the diagnosis groups was obtained by the Kruskal-Wallis test. The *p* values for all other comparisons were obtained by the Wilcoxon test. OHS: Oxford Hip Score; HHS: Harris Hip Score; OA: Osteoarthritis; ONFH: Osteonecrosis of the femoral head; RDC: Rapidly destructive coxarthrosis; UCLA: University of California Los Angeles.

**Table 6:** Clinical scores 12 months after total hip arthroplasty.

Variable		OHS Score	*p* Value	HHS Score	*p* Value	UCLA Activity Score	*p* Value
Sex	Male	15.1 ± 4.8	0.86	92.6 ± 8.0	0.37	5.7 ± 1.2	0.23
	Female	15.0 ± 3.7		94.4 ± 6.7		5.3 ± 1.1	
Diagnosis	OA	14.9 ± 4.0	0.19	94.2 ± 6.8	0.43	5.4 ± 1.2	0.22
	ONFH	15.3 ± 4.3		94.1 ± 7.3		5.5 ± 0.74	
	RDC	17.3 ± 3.0		89.3 ± 10.2		4.8 ± 0.5	
Crowe classification	Group I	14.9 ± 3.9	0.37	94.7 ± 6.3	0.0011	5.5 ± 1.1	0.22
	Group II, III, IV	15.9 ± 5.1		88.4 ± 9.0		5.1 ± 1.3	
Acetabular component	Cemented	16.5 ± 7.0	0.18	88.8 ± 11.4	0.0080	4.8 ± 2.2	0.098
	Uncemented	14.9 ± 3.9		94.3 ± 6.5		5.5 ± 1.1	
Femoral component	Cemented	15.2 ± 4.3	0.48	93.6 ± 7.4	0.29	5.3 ± 1.2	0.04
	Uncemented	14.6 ± 3.1		95.1 ± 5.2		5.8 ± 1.0	

Scores are shown as the average ± standard deviation. The *p* value for the comparison of the diagnosis groups was obtained by the Kruskal-Wallis test. The *p* values for all other comparisons were obtained by the Wilcoxon test. OHS: Oxford Hip Score; HHS: Harris Hip Score; OA: Osteoarthritis; ONFH: Osteonecrosis of the femoral head; RDC: Rapidly destructive coxarthrosis; UCLA: University of California Los Angeles.

**Table 7:** Correlation coefficients and *p* values obtained for each variable by multivariate regression analysis.

Variable	OHS at 3 Months		OHS at 6 Months		OHS at 12 Months	
	|*r*|	*p* Value	|*r*|	*p* Value	|*r*|	*p* Value
Female gender	0.19	0.011	0.46	0.96	0.36	0.14
Age	0.37	0.73	0.52	0.056	0.37	0.052
BMI	0.28	0.87	0.18	0.68	0.25	0.94
OA	0.39	0.41	0.71	0.043	0.34	0.85
Duration of surgery	0.30	0.68	0.58	0.87	0.53	0.93
Pre-op OHS	0.45	<0.001	0.49	0.018	0.19	<0.001
Pre-op HHS	0.51	0.0010	0.48	0.67	0.39	0.66
Pre-op UCLA	0.48	0.0075	0.48	0.24	0.44	0.38
Pre-op flexion ROM	0.27	0.87	0.36	0.47	0.15	0.46
Cemented cup	0.16	0.30	0.25	0.10	0.15	0.12
Cemented stem	0.36	0.47	0.45	0.012	0.40	0.36
Crowe group I	0.13	0.44	0.57	0.94	0.45	0.56
Pre-op LLD	0.14	0.71	0.43	0.58	0.25	0.54
Post-op LLD	0.11	0.39	017	0.12	0.12	0.87
Flexion at follow-up (3, 6 or 12 month)	0.41	0.82	0.50	0.53	0.32	0.60

BMI: Body mass index; OHS: Oxford Hip Score; HHS: Harris Hip Score; OA: Osteoarthritis; ONFH: Osteonecrosis of the femoral head; RDC: Rapidly destructive coxarthrosis; ROM: Range of motion; LLD: Leg length discrepancy; r: Correlation coefficient; UCLA: University of California Los Angeles.
